# Applications of different energy devices in laparoscopic and robotic gynecological surgery: a systematic review

**DOI:** 10.1007/s00404-025-08055-x

**Published:** 2025-05-27

**Authors:** Tiago Meneses Alves, Luís Ferreira De Castro, António Tomé, Hélder Ferreira

**Affiliations:** 1 Department of Gynecology and Obstetrics, Centro Materno Infantil do Norte-Unidade Local de Saúde de Santo António (CMIN-ULSSA), Porto, Portugal; 2 Instituto Ciências Biomédicas Abel Salazar-ICBAS, University of Porto, Porto, Portugal; 3 Gynecology Minimally Invasive Surgery and Endometriosis Unit, Department of Gynecology and Obstetrics, CMIN-ULSSA, Porto, Portugal

**Keywords:** Electrosurgery, Advanced energy devices, Energy source, Laparoscopy, Robotic assisted surgery, Lateral thermal spread

## Abstract

**Purpose:**

This study aims to evaluate and summarize the existing literature regarding the safety, efficacy, and outcomes of various energy sources in minimally invasive gynecological surgeries.

**Methods:**

A systematic review was conducted by searching the PubMed/MEDLINE, Cochrane Library, and Web of Science databases. We included studies that compared different energy sources used in laparoscopic and robotic gynecological surgeries, focusing on their advantages and complications. 37 studies were ultimately included in this review.

**Results:**

Among the 37 studies, 24 were randomized controlled trials, 11 were retrospective studies, and 1 was prospective. In laparoscopic procedures, advanced energy sources were associated with reduced intraoperative blood loss and shorter operative times. Specifically, ultrasonic devices demonstrated significantly less thermal damage and facilitated easier postoperative histologic assessment of lymph nodes compared to conventional electrosurgery. In robotic assisted surgeries, the literature reported shorter hospital stays and reduced thermal injury during colpotomy when utilizing laser energy. No significant differences were observed in other perioperative outcomes across both minimally invasive approaches.

**Conclusion:**

Advanced energy devices may offer advantages in gynecological minimally invasive surgery, particularly in minimizing tissue trauma and enhancing surgical precision. However, evidence on outcomes such as lateral thermal spread and tissue healing remains limited and inconsistent. Further high-quality comparative studies are needed to clarify the clinical implications of each energy source and guide optimal instrument selection, especially in robotic assisted procedures.

## Introduction

Today, most gynecological procedures utilize some form of applied energy [1]. The use of new-generation energy-based devices, particularly in minimally invasive gynecologic surgery (MIGS), such as laparoscopy or robotic procedures, is steadily increasing and has made the MIGS approach increasingly efficient and safe for the patient [2, 3]. Gynecological surgeries, regardless of their complexity, can now be performed in a less invasive manner, significantly reducing the risk of conversion to laparotomy and minimizing the likelihood of gastrointestinal or urinary injuries [4].

Monopolar (CM) and conventional bipolar (CB) electrosurgery (conventional electrosurgery modalities-CE), along with advanced bipolar devices such as LigaSure™ (LS), EnSeal™ (ENS), and Plasma Kinetic Gyrus™ (PK), as well as alternative energy sources such as ultrasonic and laser technologies, are utilized in MIGS [5, 6]. The most commonly used electrosurgical modality is monopolar energy, favored for its availability, cost-effectiveness, and versatility in tissue effects, which include desiccation, vaporization, fulguration, and coaptation [7]. Surgeons can fine-tune the type of current (cutting vs. coagulation), power output, and waveform modulation (such as pure cut, blended cut, spray coagulation, and desiccation) [6, 7]. Cutting mode typically uses a continuous, low-voltage current to achieve a clean incision with minimal hemostasis. In contrast, coagulation modes use intermittent, higher voltage currents to achieve hemostasis via tissue desiccation or fulguration [6, 7]. These settings must be selected based on the tissue type and the desired surgical effect, with caution to avoid excessive lateral thermal spread (LTS), particularly in delicate areas [6, 7]. For CB electrosurgery, power settings are generally lower compared to monopolar devices, as the current passes between two electrodes at the instrument’s tip. The tissue effect in bipolar mode is adjusted by modifying the power level, duration of activation, and tissue compression [4, 5]. Conversely, to monopolar systems, CB instruments lack waveform variation, and their effectiveness depends heavily on close and consistent tissue contact [4, 5]. In addition to the reduced versatility of tissue effects (capable of only desiccation and coaptation for vessels up to 5 mm in diameter), CB has other limitations, such as the potential for incomplete vessel sealing and the risk of LTS [4, 5]. These limitations have led to the development of new-generation bipolar devices [4, 5]. The advanced bipolar vessel sealing devices (ABDv) are powered by their own generators and utilize a tissue feedback monitoring system [1, 6]. This system can adjust the voltage based on tissue impedance to achieve the desired tissue effect while minimizing power settings and reducing the risk of LTS [1, 6]. Furthermore, these devices can seal vessels up to 7 mm in diameter (in contrast to the 5 mm limitation of CB devices, where hemostasis is achieved through thrombus formation) [6, 7]. They also feature an improved cutting system that allows tissue cutting after coagulation without additional instruments, providing a more cost-effective option for minimally invasive surgery [6, 7].

Ultrasonic instruments can achieve tissue effects similar to those produced by electrosurgical devices but without using electrosurgery principles [1, 6]. These instruments convert ultrasonic energy into mechanical and thermal energy at their jaws, allowing precise cutting control [5–7]. The latest generation of ultrasonic devices (UD) can effectively seal blood vessels up to 7 mm in diameter [5, 6]. Compared to traditional electrosurgical devices, ultrasonic technology is associated with less tissue charring, minimal LTS, and reduced smoke production [5–7]. However, surgeons should be cautious of unintended injuries that may occur if the instrument tip contacts adjacent organs, as elevated temperatures can persist for several seconds after activation [1, 6, 7]. In addition, UD may lead to slower coagulation and dissection compared to electrosurgery, and the amount of pressure applied must be carefully considered for different tissue types to achieve the desired effect, which can also lead to rapid blade fatigue [1, 6, 7].

The Thunderbeat™ (TB) device was developed to combine the effects of ultrasonic and advanced bipolar energy into a single instrument [5, 7]. Its claimed advantages include the highest median burst pressure, minimal LTS, rapid vessel sealing, and a cutting function, allowing it to seal vessels up to 7 mm in diameter [5, 7].

Laser technology was initially promoted as an alternative to electrosurgery, as it does not rely on electrical current and offers selective tissue effects with minimal LTS [7]. However, the use of lasers in gynecologic surgery has declined, primarily due to high costs and limited availability [1, 7].

Understanding the potential injuries associated with each energy source is crucial to facilitate prompt interventions if complications arise. If devices are not set up or used correctly or the surgical team does not fully understand electrosurgical principles, complications can occur [1]. According to the literature, the incidence of electrosurgery complications is approximately 2–5 per 1000 surgeries, with the most serious injuries possibly leading to fatal consequences [1, 7]. Notably, many thermal complications associated with electrosurgery can go undetected during surgery, and symptoms of bowel perforation often present about a week later, necessitating management through a laparotomy [1, 7]. Gynecologists are often perceived as lacking knowledge about the physical fundamentals of various energy sources [8]. There has been a growing body of evidence regarding these energy modalities in recent years. As a result, contemporary minimally invasive surgeons have an important responsibility to understand the principles behind these instruments. This review aims to assess the safety, efficacy, and outcomes of different energy modalities used in MIGS, focusing on the potential LTS associated with these devices.

## Materials and methods

### Sources and search strategies

A systematic literature review was conducted through a search on databases PubMed/MEDLINE, The Cochrane Library, and Web of Science identifying clinical studies published in English from January 1989 to August 2024. The following combination was used as search strategy in PubMed: (“Gynecology”[MeSH] OR “Gynecologic Surgical Procedures”[MeSH]) AND (“Minimally Invasive Surgical Procedures”[MeSH] OR “laparoscopy” OR “robotic surgery”) AND (“Monopolar electrosurgery” OR “Advanced vessel sealing device” OR “Advanced bipolar energy device” OR “Bipolar vessel sealing” OR “Vessel sealing” OR “Reusable energy devices” OR “Single-use energy device” OR “Conventional bipolar instrument” OR “Conventional bipolar electrosurgery” OR “Ultrasonic Energy” OR “Harmonic energy” OR “Sonosurg” OR “ultrasonic sealer” OR “ultrasonic coagulating shears” OR “Thunderbeat” OR “Energy devices” OR “Bipolar electrosurgery” OR “EnSeal” OR “Gyrus” OR “LigaSure” OR “Laser technology” OR “PlasmaKinetic” OR “Argon plasma coagulation”). Equivalent search strategies were adapted for the Cochrane Library and Web of Science using appropriate syntax and controlled vocabulary where applicable. All the screening and exclusion processes were realized by two independent reviewers (T.M.A. and L.F.D.C.) based on the Preferred Reporting Items for Systematic Reviews and Meta-Analyses (PRISMA) 2020 guidelines. In addition, a detailed revision through the references of all the studies included was made to find other eligible publications.

### Inclusion and exclusion criteria

Our research included randomized controlled trials (RCT) and observational studies (prospective or retrospective). The selection was based on the PICO search strategy. Population and intervention: women who underwent laparoscopic or robotic surgery for gynecological pathology, including laparoscopically assisted vaginal hysterectomy (LAVH) and via transvaginal natural orifice transluminal endoscopic surgery (NOTES); comparison: at least between two energy sources; outcomes: estimated blood loss, operative time, postoperative pain, hospital stay length, complications (namely LTS and other energy source-related injuries). At least one of the outcomes of interest had to be reported on the article to be selected for our review.

Hysteroscopic studies, laboratory and animal studies, and investigations that did not compare two or more energy modalities in laparoscopic or robotic gynecological surgery were excluded.

### Study inclusion and data collection

All the titles and abstracts of included studies were screened independently by two authors (T.M.A. and L.F.D.C.). Posteriorly, another review team member (H.F.) independently assessed the full text of the initial studies selected for eligibility. A standardized protocol was created and used to collect data from the final publications selected: characteristics of participants included (age, diagnosis, reason for laparoscopic/robotic procedure and type of surgery, number the participants and per group studied), types of energy source compared, outcomes of interest, and key findings. Two authors (T.M.A. and L.F.D.C.) extracted the data from the selected studies. Any disagreement over the data extracted was first discussed between these authors. If a consensus could not be reached, a third author (H.F.) was consulted to resolve disagreements through discussion until mutual agreement was obtained.

### Quality assessment

According to the Cochrane risk-of-bias tool, two authors (T.M.A. and L.F.D.C.) independently assessed the risk of bias of randomized (RoB 2) and non-randomized (ROBINS-I) studies included in this systematic review [9, 10]. A third author (H.F.) resolved any disagreement related to the risk-of-bias assessment.

## Results

**Fig. 1:**
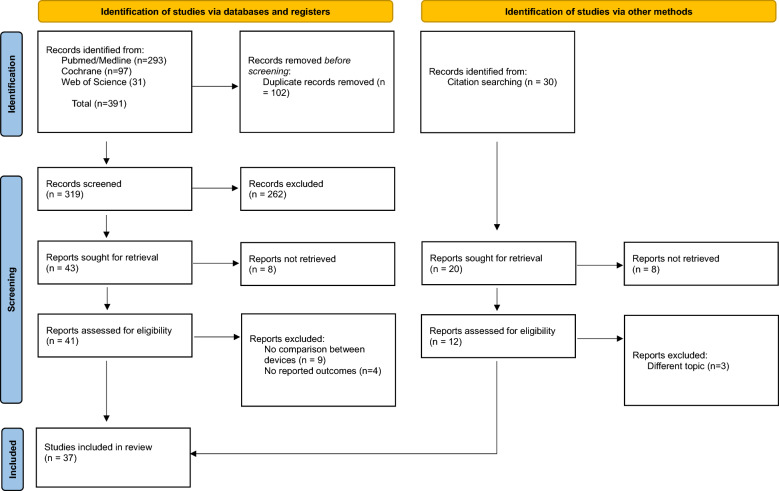
PRISMA flowchart summarizing article selection

Our systematic literature review identified 421 papers, of which 30 were retrieved from the references of the identified publications in the databases. After removing duplicates, we conducted a screening of titles and abstracts. This process led to the selection of 53 potentially relevant publications. Applying our inclusion and exclusion criteria, we excluded 16 studies, resulting in a final count of 37 studies in this systematic review (Fig. 1). Among these 37 studies, 24 were RCTs, and 13 were non-RCTs. All the studies examined various classes of energy sources used in either laparoscopic (*N* = 34) or robotic (*N* = 3) gynecological surgery. Twenty-four laparoscopic studies compared standard electrosurgery with either UD or advanced bipolar energy. Six studies directly compared the devices within the latter category, and four only compared ultrasonic energy with advanced bipolar energy. In robotic surgery, two studies compared laser technology with ultrasonic instruments, and one compared monopolar electrosurgery to ultrasonic energy.

Of the 37 included manuscripts, 6 were related to malignant gynecological conditions [11–16]. The final eligible studies were published between 2000 [17] and 2024 [18], with a sample size ranging from 20 [19] to 817 [20] women.

The primary endpoints of all studies focused on comparing perioperative outcomes between two different energy sources. However, one prospective single-center trial aimed primarily to evaluate the feasibility of laser angiography during a robot-assisted total laparoscopic hysterectomy to assess vascular perfusion of the vaginal cuff [19].

Table 1 summarizes the outcomes and key findings of the included studies.

**Table 1:** Comparative studies on energy devices used in laparoscopic and robotic gynecology surgery

Authors, year	Study design	Devices compared	Study cohort	Procedure	Blood loss (mL)	Mean operative time (min)	Postoperative pain score	Hospital stay (days)	Complications (*n*)	Lateral thermal spread: distance (mm)	Significant results
Holub et al., 2000	Randomized controlled trial	HS vs. CE (monopolar: scissors)	*N* = 70 (HS: 46, CE: 34)	Laparoscopic hysterectomy	HS: 166 CE: 170 (*p* > 0.05)	HS: 82.9 CE: 90.6 (*p* = 0.24)	NA	HS: 3.1 CE: 3.3 (*p* > 0.05)	No statistically significant difference	NA	No statistically significant difference was found between both devices
Holub et al., 2001	Prospective randomized comparative study	HS vs. CE	*N* = 30 (US: 15, CE: 15)	TLH + LND	HS: 110 CE: 150 (*p* = 0.91)	HS: 138.3 CE: 132.1 (*p* = 0.96)	NA	HS: 3.7 CE: 4.3 (*p* = 0.23)	No statistically significant difference	NA	The number of lymph nodes harvested was significantly higher in HS group compared to CE group (18 vs 12.7, respectively; *p* = 0.05)
Holub et al., 2002	Retrospective comparative study	CE (monopolar: dissector) vs. HS	*N* = 59 (CE: 32, HS: 27)	LAVH + BSO + LND	CE: 210.2 HS: 194.2 (*p* > 0.05)	CE: 148.2 HS: 155.1 (*p* > 0.05)	NA	CE: 4.2 HS: 3.6 (*p* = NS)	No statistically significant difference	HS caused less thermal damage (mm not mentioned)	The number of lymph nodes harvested was significantly higher in HS group compared to CE group (17.5 vs 13.7, respectively; *p* = 0.0008)
Ou et al., 2004	Retrospective comparative study	CB vs. PK	*N* = 123 (CB: 73, PK: 50)	TLH	CB: 172.1 PK: 111.8 (*p* = 0.021)	CB: 65.8 PK: 64.8 (*p* = 0.89)	NA	NA	No statistically significant difference	NA	Surgeries performed with PK were associated with significantly less blood loss compared to those using CB
Wang et al., 2005	Prospective comparative study	CE vs. PK	*N* = 62 (CE: 31, PK: 31)	LAVH	CE: 253.2 PK: 196.8 (*p* = 0.105)	CE: 93.4 PK: 87.6 (*p* = 0.368)	NA	CE: 3.0 PK: 3.2 (*p* = 0.499)	No statistically significant difference	NA	No significant difference was found between CE and PK
Holub et al., 2005	Retrospective comparative study	CE (monopolar: dissector) vs. HS	*N* = 119 (CE: 37, HS: 82)	Laparoscopic hysterectomy ± LND	CE: 205.3 HS: 188.6 (*p* > 0.05)	CE: 144.2 HS: 158.7 (*p* > 0.05)	NA	CE: 4.2 HS: 3.4 (*p* > 0.05)	No statistically significant difference	NA	The number of lymph nodes harvested was significant higher in HS group compared to CE group (18.1 vs 13.7, respectively; *p* < 0.001)
Lee et al., 2007	Retrospective case–control study	PK vs. CB	*N* = 76 (PK: 38, CB: 38)	LRH + LND	PK: 397.4 CB: 564.5 (*p* < 0.03)	PK: 171.8 CB: 228.9 (*p* < 0.0001)	NA	PK: 7.5 CB: 6.9 (*p* = 0.1)	Complications within 60 days after surgery: PK: 0 vs. CB: 5 (2 intestinal obstruction, 2 acute renal failure, 1 vesicovaginal fistula) (*p* < 0.01)	NA	Compared to CE for performing LRH, PK was associated with significantly shorter operative time, reduced blood loss, and fewer postoperative complications
Demirturk et al., 2007	Retrospective study	LS vs. HS	*N* = 40 (LS: 21, HS: 19)	TLH	LS: 87.76 HS: 152.63 (*p* < 0.001)	LS: 59.57 HS: 90.95 (*p* < 0.001)	NA	LS: 3.24 HS: 3.42 (*p* = 0.436)	NA	NA	LS was significantly associated with shorter operative time and reduce intraoperative bleeding compared to HS
Litta et al., 2010	Randomized controlled study	CE (monopolar: needle) vs. HS	*N* = 160 (CE: 80, HS: 80)	LM	Intraoperative blood loss: CE: 182.8 HS: 135.2 (*p* = 0.004) Total blood loss ($\delta$ Hb): CE: 1.2 HS: 0.9 (*p* = 0.03)	CE: 88.8 HS: 71.8 (*p* = 0.000)	24 h after surgery: CE: 5.6 HS: 4.4 (*p* = 0.00)	CE: 2.76 HS: 2.27 (*p* = 0.00)	No statistically significant difference	NA	HS resulted in significantly shorter overall operative time and was associated with reduced blood loss and postoperative pain compared to CE
Janssen et al., 2011	Randomized controlled trial	LS vs. CB	*N* = 140 (LS: 70, CB: 70)	Laparoscopic hysterectomy	LS: 234.1 CB: 273.1 (*p* = 0.46)	LS: 148.2 CB: 142.1 (*p* = 0.46)	NA	LS: 2.9 CB: 2.9 (*p* = 0.94)	No statistically significant difference	NA	No significant differences in operative time or blood loss were identified between LS and CB in laparoscopic hysterectomy
Hsuan su et al., 2011	Retrospective case–control study	PK vs. CE	*N* = 194 (PK: 97, CE: 97)	LM	PK: 190.4 CE: 243.8 (*p* < 0.025)	PK: 117.8 CE: 116.9 (*p* = 0.906)	NA	PK: 2.7 CE: 2.8 (*p* = 0.315)	No statistically significant difference	NA	PK showed significantly less blood loss in LM when compared to CE
Janssen et al., 2012	Randomized controlled trial	LS vs. CB	*N* = 100 (LS: 51, CB: 49)	Laparoscopic salpingo-oophorectomy (8 unilateral, 92 bilateral)	LS: 38.0 CB: 33.3 (*p* = 0.73)	LS: 54.6 CB: 58.6 (*p* = 0.46)	NA	LS: 1.3 CB: 1.2 (*p* = 0.89)	No statistically significant difference	NA	LS is at least reliable as CB during laparoscopic salpingo-oophorectomy (no significant differences was observed in outcomes between both devices)
Cho et al., 2012	Retrospective case–control study	PK vs. CB	*N* = 80 (PK: 40, CB: 40)	TLH	PK: 467.9 CB: 515..3 (*p* < 0.05)	PK: 157.3 CB: 173.4 (*p* < 0.05)	NA	PK: 6.5 CB: 6.2 (*p* > 0.05)	No statistically significant difference	NA	PK was associated with significantly reduced blood loss and shorter operative time during TLH compared to CB
Ashraf Ta et al., 2012	Randomized clinical trial	HS vs. LS	*N* = 40 (HS: 20, LS: 20)	LASH	Hemoglobin drop (%): HS: 3.15 LS: 0.43 (*p* < 0.005)	HS: 138.25 LS: 64.15 (*p* < 0.005)	NA	HS: 2.00 LS: 1.65 (*p* = 0.354)	No statistically significant difference	NA	In LASH, LS resulted in significantly less blood loss and shorter operative time compared to HS
Rothmund et al., 2013	Randomized controlled trial	ENS vs. CB	*N* = 160 (ENS: 80, CB: 80)	LASH	BL < 50 mL: ENS (* N* = 72) vs. CB (*N* = 62) (*p* = 0.03) BL 50–100 mL: ENS (* N* = 8) vs. CB (* N* = 18) (*p* < 0.001)	ENS: 78.18 vs. CB: 86.30 (*p* = 0.03)	No statistically significant difference (at 24 h, 48 h and 72 h)	ENS: 2.01 vs. CB: 2.17 (*p* = 0.03)	No statistically significant difference	NA	ENS was associated with shorter total operative time, less blood loss and hospital stay. This device was at least as reliable as the conventional bipolar coagulation forceps to perform LASH
Rothmund et al., 2013	Randomized controlled trial	HS vs. BiCision	*N* = 60 (HS: 30 BC: 30)	LASH	Intraoperative BL (score): HS: 1.63 $\pm$ 0.49 BiCision: 1.07 $\pm 0.25$ (*p* < 0.0001)	Preparation time per side (mean): HS: 8.3 BiCision: 8.8 (*p* = 0.31)	NA	NA	No complications were observed for both devices	NA	BiCision is at least as reliable as HS for routine gynecological laparoscopic procedures and demonstrates reduced blood loss, improved tissue fixation, and less tissue sticking compared to HS
Fagotti et al., 2014	Randomized controlled trial	TB vs. CE (monopolar: scissors)	*N* = 50 (TB: 25, CE: 25)	LRH + LND	TB: 50; CE: 50 (*p* = 0.52)	TB: 85; CE: 115 (*p* = 0.001)	24 h after surgery: at rest: TB: 1.96; CE: 3.35 (*p* = 0.005) After Valsalva maneuver: TB: 3.17; CE: 4.65 (*p* = 0.008)	TB: 3 CE: 3 (*p* = 0.82)	No statistically significant difference	NA	LRH with LND was performed in a shorter time and less postoperative pain when using TB compared to CE
Aytan et al., 2014	Randomized prospective study	LS vs. ENS vs. PK	*N* = 45 (LS: 15, ENS: 15, PK: 15)	TLH	LS: 138.0; ENS: 218.0; PK: 118.0 (*p* = 0.004)	LS: 52.4; ENS: 55.7; PK: 51.9 (*p* = 0.73)	NA	LS: 1.1; ENS: 1.4; PK: 1.2 (*p* = 0.22)	No statistically significant difference	NA	Both devices had similar results in TLH except ENS group had more blood loss
Billow et al., 2014	Randomized prospective study	CM vs. CO_2_ laser	*N* = 21 (CM: 11, CO_2_ laser: 10)	Colpotomy during RATLH	NA	NA	NA	NA	NA	Assessment with H&E staining: CO_2_ laser: 0.7 MP: 1.1 (*p* = 0.0191)	CO_2_ laser resulted in less lateral damage compared to CM
Bansal et al., 2014	Randomized clinical trial	LS vs. HS	*N* = 242 (LS: 121, HS: 121)	TLH	LS: 88.74 HS: 140.84 (*p* < 0.005)	LS: 76.16 HS: 115.35 (*p* < 0.005)	NA	LS: 1.55 HS: 1.13 (*p* > 0.005)	No statistically significant difference	NA	LS was less time-consuming and caused less blood loss compared to HS
Choussein et al., 2015	Retrospective cohort study	CO_2_ laser vs. HS	*N* = 236 (CO_2_ laser: 85, HS: 151)	RALM	CO_2_ laser: 96.2 HS: 180.7 (*p* = 0.95)	CO_2_ laser: 182.2 HS: 195.9 (*p* = 0.55)	NA	CO_2_ laser: 0.15 HS: 0.64 (*p* = 0.004)	No statistically significant difference	NA	CO_2_ laser was at least reliable as HS for RALM
Holloran-Schwartz et al., 2016	Randomized controlled trial	LS vs. CB	*N* = 46 (LS: 24, CB: 22)	TLH	75 (not compared between devices)	Time to desiccation and transection of each side of uterus attachments: LS: 8.4 CB: 14.6 (*p* < 0.001)	NA	NA	No statistically significant difference	NA	LS has reduced operative time and total intraoperative direct costs compared to CB
Kuo et al., 2017	Retrospective comparative study	HS vs. CE	*N* = 124 (HS: 31, CE: 93)	LM	HS: 300.0 CE: 214.7 (*p* = 0.063)	HS: 119.7 CE: 106.0 (*p* = 0.154)	NA	HS: 2.0 CE: 2.5 (*p* < 0.001)	HS: 0 vs. CE: 4 (2 low-grade fever, 1 urinary tract infection, 1 subcutaneous ecchymosis at the port site) (*p* = 0.023)	NA	HS was associated with a shorter hospital stay but incurred higher hospital costs compared to CE
Shiber et al., 2018	Randomized controlled trial	LS vs. ENS	*N* = 140 (LS: 70, ENS: 70)	TLH	LS: 100 ENS: 100 (*p* = 0.5823)	Time to bilateral uterine arteries ligation: LS: 30 ENS: 35 (*p* = 0.0281); Total operative time: LS: 85 ENS: 97 (*p* = 0.0821)	NA	NA	No statistically significant difference	NA	ENS was associated with significantly higher rates of device failure (*p* = 0.003), increased surgeon-perceived workload (*p* < 0.0001), and a longer time to achieve bilateral ligation of the uterine arteries."
Hasanov et al., 2018	Randomized controlled trial	LS vs. MS	*N* = 74 (LS: 37; MS: 37)	TLH	LS: 164; MS: 160 (*p* = 0.36)	Time to uterine arteries ligation: LS: 22.7; MS: 26.4 (*p* = 0.89)	LS: 1; MS: 0 (*p* = NA)	LS: 4 MS: 4 (*p* = NA)	LS: 0; MS: 4 (*p* = NA)	NA	MS was at least as reliable as LS, particularly in terms of mean operative time and estimated intraoperative blood loss
Taşkin et al., 2018	Randomized controlled trial	LS vs. CB	*N* = 68 (LS: 34; CB: 34)	TLH + retroperitoneal LND	LS: 176.1; CB: 182.3 (*p* = 0.783)	LS: 134.2; CB: 163.5 (*p* < 0.001)	8 h after surgery: LS: 3.3; CB: 3.6 (*p* = 0.278) 24 h after surgery: LS: 2.1; CB: 2.1 (*p* = 1.0)	LS: 1.9 CB: 2.1 (*p* = 0.48)	No statistically significant difference	NA	LS and CB revealed comparable perioperative outcomes except for operative time that was shorter with LS
Choi et al., 2018	Randomized controlled trial	HS vs. CM (monopolar: scissors)	*N* = 40 (HS: 20; CM: 20)	Colpotomy during TLH	HS: 51.4 CM: 46.0 (*p* = 0.820)	Colpotomy: HS: 7.2; MD: 4.1 (*p* < 0.001) Operative time: HS: 68 CM: 59 (*p* = 0.081)	NA	HS: 2.2 CM: 2.3 (*p* = 0.799)	No statistically significant difference	HS: 950 µm MD: 1500 µm (*p* = 0.037)	HS showed better laparoscopic visibility and caused significantly less lateral thermal damage during colpotomy compared to CM
Beran et al., 2018	Randomized controlled trial	CM (monopolar: scissors) vs. HS	*N* = 20 (CM: 10, HS: 10)	Colpotomy during RATLH	62.5 (not compared between devices)	NA	NA	NA	NA	No statistically significant difference was observed in judge perfusion before or after cuff closure between both devices	Laser angiography can be a feasible tool to evaluate vaginal cuff perfusion during RATLH
Li et al., 2018	Retrospective study	LS vs. CB	*N* = 756 (LS: 225, CB: 531)	LM	LS: 182.62 CB: 212.99 (*p* = 0.156)	LS: 109.09 CB: 114.44 (*p* = 0.268)	NA	LS: 2.10 CB: 2.57 (*p* < 0.001)	No statistically significant difference	NA	LS was associated with a significantly shorter hospital stay. However, CB proved to be significantly more efficient for small and medium-sized myomas and was associated with lower hospital costs
Huang et al., 2018	Retrospective study	CE vs. LS vs. HS	*N* = 817 (CE: 481, LS: 256, HS: 80)	LM	CE: 175.4 LS: 201.0 HS: 245.8 (*p* = 0.003)	CE: 100.1 LS: 115.7 HS: 130.8 (*p* < 0.001)	NA	CE: 2.5 LS: 2.1 HS: 2.0 (*p* < 0.001)	No statistically significant difference	NA	The LS and HS groups experienced significantly greater blood loss and longer operative times; however, both groups had a higher number and larger size of fibroids removed compared to the CE group. In addition, hospital stays were significantly shorter in the LS and HS groups
Yuksel et al., 2019	Randomized controlled trial	LS vs. ENS	*N* = 132 (LS: 67, ENS: 65)	TLH	LS: 128.2; ENS: 110.1 (*p* = 0.295)	Mean operative time (time from transection of round ligament to colpotomy): LS: 25.7; ENS: 38.2 (*p* = 0.001) Total operative time: LS: 92.3 ENS: 95.1 (*p* = 0.360)	NA	NA	No statistically significant difference	NA	LS had statistically significant shorter mean time from transection of round ligament to colpotomy compared to ENS
Lee et al., 2019	Randomized controlled trial	LS vs. CB	*N* = 71 (LS: 36, CB: 35)	Hysterectomy via transvaginal NOTES	LS: 269.23 CB: 310.60 (*p* = 0.445)	LS: 88.58 CB: 99.54 (*p* = 0.063)	At 24 h after surgery: LS: 3.9 CB: 2.5 (*p* = 0.006) At 36 h after surgery: LS: 2.8, CB: 1.4 (*p* = 0.002) At 48 h after surgery: LS: 1.3, CB: 1.0 (*p* = 0.313)	LS: 3.34 CB: 3.37 (*p* = 0.858)	No statistically significant difference	NA	LS is a feasible and safe device and had significantly reduced operative time for hysterectomy via transvaginal NOTES only (without additional procedures, e.g., salpingo-oophorectomy or adhesiolysis) compared to CB (*p* = 0.029)
Wong et al., 2020	Randomized controlled trial	LS vs. PK	*N* = 64 (LS: 31, PK: 33)	TLH	LS: 50 PK: 50 (*p* = 0.84)	LS: 63.8; PK: 74.4 (*p* = 0.03)	No statistically significant difference	LS: 3; PK: 3 (*p* = 0.37)	No statistically significant difference	NA	LS was statistically significantly faster to achieve hemostasis during TLH than PK
Talwar et al., 2021	Prospective randomized case–control study	ALAN vs. ENS	*N* = 100 (ALAN: 50, ENS: 50)	TLH	ALAN: 111.40 ENS: 107.84 (*p* = 0.4)	ALAN: 56.90 ENS: 57.25 (*p* = 0.9)	NA	NA	No statistically significant difference	NA	ALAN was at least as reliable as ENS in TLH but more cost-effective
Batra et al., 2022	Randomized controlled trial	CB vs. LS	*N* = 120 (CB: 60, LS: 60)	TLH	CB: 145 LS: 141.67 (*p* = 0.846)	CB: 142.50 LS: 136.37 (*p* = 0.002)	NA	CB: 2.54 LS: 2.32 (*p* = 0.128)	No statistically significant difference	NA	LS was a reliable device with shorter operative times compared to CB
Hasabe et al., 2023	Randomized controlled trial	HS vs. LS vs. bipolar shearer	*N* = 90 (HS: 30, LS: 30, Bipolar Shearer: 30)	TLH	$\delta$ Hb (%) HS: 2.15 LS: 1.26 Bipolar shearer: 1.54	HS: 68.25 LS: 54.36 Bipolar shearer: 59.34	NA	HS: 1.84 LS: 1.35 Bipolar shearer: 1.60	Only related to intraoperative blood loss: HS: 6 LS: 4 bipolar Shearer: 5	NA	HS resulted in more blood loss and larger operative time compared to LS and bipolar shearer
Gorginzadeh et al., 2024	Randomized controlled trial	CM (monopolar: hook) vs. HS	*N* = 78 (CM: 39, HS: 39)	Colpotomy during TLH	CM: 63.81 HS: 36.71 (*p* = 0.477)	Colpotomy duration: CM: 8.47 HS: 9.97 (*p* = 0.493) Total operative time: CM: 132.95 HS: 119.00 (*p* = 0.160)	No statistically significant difference	NA	No statistically significant difference	HS resulted in statistically less lateral thermal damage in the right border of the cervix: HS: 3.08; CM: 3.85 (*p* = 0.001)	No significant difference was found between CM and HS except that HS was associated with significantly less tissue injury in the right lateral cuff area during colpotomy

*BSO* bilateral salpingo-oophorectomy, *CB* conventional bipolar, *CE* conventional electrosurgery, *CM* conventional monopolar, *ENS* EnSeal, *H&*E hematoxylin and eosin, HS harmonic scalpel, *LAVH* laparoscopically assisted vaginal hysterectomy, *LASH* laparoscopic supracervical hysterectomy, *LND* lymph node dissection, *LRH* laparoscopic radical hysterectomy, *LS* LigaSure, *MS* MarSeal, *NA* not applicable/assessed/available,* NOTES* natural orifice transluminal endoscopic surgery, *PK* plasmakinetic system,* RALM* robot-assisted laparoscopic myomectomy, *RATLH* robot-assisted total laparoscopic hysterectomy, *TB* thunderbeat,* TLH* total laparoscopic hysterectomy

Table 1 comparative studies on energy devices used in laparoscopic and robotic gynecology surgery.

### Laparoscopic approach

#### Device type

Twenty-five out of the 37 studies reported data on ABDv. Our literature review found several studies comparing different ABDv to Harmonic Scalpel™ (HS) [21–23] or CB energy [13, 24]. Regardless of the type of gynecological surgery, most of these studies showed that ABDv were associated with lower blood loss (BL), shorter operating times, and hospital stay compared to other energy sources [21–23, 25, 26]. LS was the most ABDv evaluated and was effective in the various perioperative outcomes studied (Table 1).

Several other studies compare the efficacy of UD to other energy instruments. Indeed, a few studies showed the superiority of UD to CE. For laparoscopic staging of endometrial and cervical cancer, two retrospective studies [12, 15] and one prospective study [14] noted a significantly higher number of lymph nodes harvested in the HS group compared to the CE group (17.5 vs. 13.7, *p* = 0.0008; 18.1 vs. 13.7, *p* < 0.001 and 18 vs 12.7, *p* = 0.05, respectively). In addition, two studies on laparoscopic myomectomy (LM) showed significantly shorter global operative time, less intraoperative BL, postoperative pain score, and a shorter hospital stay than CE [27, 28]. However, HS was associated with higher hospital expenses [28]. In addition, two RCTs showed significantly less lateral thermal damage with HS during colpotomy when compared to monopolar energy [18, 29].

Fagotti et al., in a randomized controlled trial, reported that laparoscopic radical hysterectomy (LRH) with lymph node dissection (LND) was performed in a shorter operative time with fewer postoperative pain scores using TB compared to CE [11].

#### Surgical procedure

The laparoscopic hysterectomy (LH) was the principal surgery where the various properties of energy sources available for gynecological laparoscopic surgery were investigated. For LH, ABDv were reported to be associated with significantly less BL, shorter operative times, and less total intraoperative direct costs than ultrasonic and conventional devices, as stated in Table 2 [21, 23–25, 30–32]. Pooled mean operative times for LH using ALAN were lowest (56.90 min $\pm$ 12.45) as well as the pooled mean BL and hospital stay using ENS (98.59 min $\pm$ 12.45 76.3 and 1.4 $\pm$ 0.5, respectively) when compared to the other devices. However, three studies were excluded from the pooled analysis due to significant heterogeneity in LH outcome assessment methods [26, 32, 33].

**Table 2:** Summary statistics of perioperative outcomes by energy device type and procedure

Procedures/perioperative outcomes	Energy sources (pooled mean/standard deviation)										
	CM	CB	CE	LS	PK	ENS	ALAN	MS	TB	HS	CO_2_ laser
Laparoscopic hysterectomy											
Blood loss (mL)	NA	193.52 $\pm$ 184.25 (*n* = 243)	170 $\pm$ 87.5 (*n* = 34)	100.2 $\pm$ 124.6 (*n* = 502)	200.91 $\pm$ 42.02 (*n* = 138)	98.59 $\pm$ 76.3 (*n* = 200)	114.40 $\pm$ 22.32 (*n* = 50)	160 $\pm$ 21.75 (*n* = 37)	NA	148.25 $\pm$ 47.06 (*n* = 186)	NA
Mean operative time (min)	NA	118.48 $\pm$ 29.06 (*n* = 243)	90.6 $\pm$ 30 (*n* = 34)	112.53 $\pm$ 61.43 (*n* = 395)	107.01 $\pm$ 48.03 (*n* = 138)	83.32 $\pm$ 32.6 (*n* = 200)	56.90 $\pm$ 12.45 (*n* = 50)	NA	NA	105.38 $\pm$ 22.39 (*n* = 186)	NA
Hospital stay (days)	NA	3.55 $\pm$ 1.07 (*n* = 170)	3.3 $\pm$ 1.25 (*n* = 34)	2.24 $\pm$ 0.98 (*n* = 318)	4.28 $\pm$ 2.28 (*n* = 88)	1.4 $\pm$ 0.5 (*n* = 15)	NA	4 $\pm$ 0.25 (*n* = 37)	NA	1.85 $\pm$ 0.79 (*n* = 186)	NA
Laparoscopic supracervical hysterectomy											
Blood loss (mL)	NA	NA^a^	NA	NA^a^	NA	NA^a^	NA	NA	NA	NA^a^	NA
Mean operative time (min)	NA	86.3 $\pm$ 35.34 (*n* = 80)	NA	64.15 $\pm$ 12.02 (*n* = 20)	NA	78.18 $\pm$ 33.96 (*n* = 80)	NA	NA	NA	138.25 $\pm$ 23.41 (*n* = 20)	NA
Hospital stay (days)	NA	2.17 $\pm$ 0.47 (*n* = 80)	NA	2.17 $\pm$ 0.47 (*n* = 20)	NA	2.01 $\pm$ 0.44 (*n* = 80)	NA	NA	NA	2.0 $\pm$ 1.52 (*n* = 20)	NA
Laparoscopically assisted vaginal hysterectomy											
Blood loss (mL)	NA	NA	231.5 $\pm$ 141.96 (*n* = 63)	NA	196.8 $\pm$ 143.7 (*n* = 31)	NA	NA	NA	NA	194.2 $\pm$ 15 (*n* = 27)	NA
Mean operative time (min)	NA	NA	121.3 $\pm$ 53.45 (*n* = 63)	NA	87.6 $\pm$ 28.1 (*n* = 31)	NA	NA	NA	NA	155.1 $\pm$ 16.25 (*n* = 27)	NA
Hospital stay (days)	NA	NA	3.61 $\pm$ 1.66 (*n* = 63)	NA	3.2 $\pm$ 1.0 (*n* = 31)	NA	NA	NA	NA	3.6 $\pm$ 1.25 (*n* = 27)	NA
Hysterectomy via transvaginal notes											
Blood loss (mL)	NA	310.60 $\pm$ 220.60 (*n* = 35)	NA	269.23 $\pm$ 232.47 (*n* = 36)	NA	NA	NA	NA	NA	NA	NA
Mean operative time (min)	NA	99.54 $\pm$ 31.96 (*n* = 35)	NA	88.58 $\pm$ 30.21 (*n* = 36)	NA	NA	NA	NA	NA	NA	NA
Hospital stay (days)	NA	3.37 $\pm$ 0.77 (*n* = 35)	NA	3.34 $\pm$ 0.54 (*n* = 36)	NA	NA	NA	NA	NA	NA	NA
Laparoscopic hysterectomy + lymph node dissection											
Blood loss (mL)	NA	384.01 $\pm$ 471.01 (*n* = 72)	144.1 $\pm$ 170.6 (*n* = 77)	176.1 $\pm$ 78.2 (*n* = 34)	397.4 $\pm$ 275 (*n* = 38)	NA	NA	NA	50 $\pm$ 57.5 (*n* = 25)	176.44 $\pm$ 95.45 (*n* = 97)	NA
Mean operative time (min)	NA	198.01 $\pm$ 56.31 (*n* = 72)	132.37 $\pm$ 35.8 (*n* = 77)	134.2 $\pm$ 29.7 (*n* = 34)	171.8 $\pm$ 50.5 (*n* = 38)	NA	NA	NA	85 $\pm$ 25 (*n* = 25)	155.54 $\pm$ 28.84 (*n* = 97)	NA
Hospital stay (days)	NA	4.63 $\pm$ 3.45 (*n* = 72)	3.83 $\pm$ 1.28 (*n* = 77)	1.9 $\pm$ 0.9 (*n* = 34)	7.5 $\pm$ 2.5 (*n* = 38)	NA	NA	NA	3 $\pm$ 0.75 (*n* = 25)	2.35 $\pm$ 1.26 (*n* = 97)	NA
Colpotomy (total laparoscopic hysterectomy)											
Blood loss (mL) (during all TLH)	57.74 $\pm$ 27.12 (*n* = 59)	NA	NA	NA	NA	NA	NA	NA	NA	41.67 $\pm$ 29.04 (*n* = 59)	NA
Mean operative time (min)	6.99 $\pm$ 1.44 (*n* = 59)	NA	NA	NA	NA	NA	NA	NA	NA	9.03 $\pm$ 2.33 (*n* = 59)	NA
Hospital stay (days)	2.3 $\pm$ 0.6 (*n* = 59)	NA	NA	NA	NA	NA	NA	NA	NA	2.2 $\pm$ 0.5 (*n* = 59)	NA
Lateral thermal spread: distance (mm)	254.76 $\pm$ 8.25 (*n* = 59)	NA	NA	NA	NA	NA	NA	NA	NA	204.47 $\pm$ 2.61 (*n* = 59)	NA
Laparoscopic myomectomy											
Blood loss (mL)	NA	212.99 $\pm$ 215.7 (*n* = 531)	189.8 $\pm$ 172.6 (*n* = 751)	192.44 $\pm$ 171.33 (*n* = 481)	190.4 $\pm$ 178.5 (*n* = 97)	NA	NA	NA	NA	208.3 $\pm$ 184.26 (*n* = 191)	NA
Mean operative time (min)	NA	114.44 $\pm$ 51.66 (*n* = 531)	101.8 $\pm$ 43.1 (*n* = 751)	111.75 $\pm$ 42.06 (*n* = 481)	117.8 $\pm$ 37.1 (*n* = 97)	NA	NA	NA	NA	104.3 $\pm$ 47.74 (*n* = 191)	NA
Hospital stay (days)	NA	2.57 $\pm$ 0.91 (*n* = 531)	2.57 $\pm$ 0.83 (*n* = 751)	2.10 $\pm$ 0.54 (*n* = 481)	2.7 $\pm$ 0.7 (*n* = 97)	NA	NA	NA	NA	2.11 $\pm$ 0.52 (*n* = 191)	NA
Robot-assisted laparoscopic myomectomy											
Blood loss (mL)	NA	NA	NA	NA	NA	NA	NA	NA	NA	180.7 $\pm$ 218.13 (*n* = 151)	96.2 $\pm$ 115 (*n* = 85)
Mean operative time (min)	NA	NA	NA	NA	NA	NA	NA	NA	NA	195.9 $\pm$ 56.5 (*n* = 151)	182.2 $\pm$ 60.3 (*n* = 85)
Hospital stay (days)	NA	NA	NA	NA	NA	NA	NA	NA	NA	0.64 $\pm$ 1.31 (*n* = 151)	0.15 $\pm$ 0.39 (*n* = 85)
Salpingo-oophorectomy											
Blood loss (mL)	NA	33.3 $\pm$ 65..7 (*n* = 49)	NA	38 $\pm$ 69 (*n* = 51)	NA	NA	NA	NA	NA	NA	NA
Mean operative time (min)	NA	58.6 $\pm$ 23.2 (*n* = 49)	NA	54.6 $\pm$ 29.6 (*n* = 51)	NA	NA	NA	NA	NA	NA	NA
Hospital stay (days)	NA	1.2 $\pm$ 1.5 (*n* = 49)	NA	1.3 $\pm$ 2.2 (*n* = 51)	NA	NA	NA	NA	NA	NA	NA

*CB* conventional bipolar, *CE* conventional electrosurgery, *CM* conventional monopolar, *ENS* EnSeal, *HS* harmonic scalpel, *LND* lymph node dissection, *LS* LigaSure, *MS* MarSeal, *NA* not applicable/assessed/available, *NOTES* natural orifice transluminal endoscopic surgery, *PK* plasmakinetic system, *TB* thunderbeat

^a^Heterogeneous outcome assessments across studies precluded pooled analysis

Significantly less BL, shorter operative time, and hospital stay were also observed with the use of ABDv such as LS, ENS, and BiCision in laparoscopic supracervical hysterectomy (LASH) when compared to UD or CB [22, 26, 34].

Few studies evaluated the superiority of one device over another in LAVH [11, 12, 14, 15]. Pool mean BL, operative times, and hospital stay for LAVH using PK were the lowest compared to CE and HS, as shown in Table 2.

In the RCT of Lee et al. [35], 71 patients were thoroughly analyzed comparing LS to a CB instrument in hysterectomy via transvaginal NOTES, and postoperative pain scores were significantly lower in the conventional device group up to the first 36 h (at 24 h: 3.9 vs. 2.5, *p* = 0.006; at 36 h: 2.8 vs. 1.4, *p* = 0.002). However, LS had significantly reduced operative time for hysterectomy (without additional procedures such as salpingo-oophorectomy or adhesiolysis).

Regarding LM, the pooled data from 5 studies [20, 27, 28, 36, 37] showed that CE devices had shorter operative times (101.8 min; $\sigma$ $\pm$ 43.1) and less BL (189.8 mL; $\sigma \pm$ 172.6) when compared to other type of energy devices. When comparing hospital stay days across all studies in LM, LS yielded a shorter hospital stay of 2.10 days compared with the opposite extreme of 2.7 days with PK.

One RCT on laparoscopic salpingo-oophorectomy [38] reported no superiority of LS to CB, with no significant differences in outcomes between both devices.

#### Perioperative outcomes

##### Operative time

An important aspect of energy sources in laparoscopy is the need to reduce operating times while maintaining adequate hemostasis, which is often cited as an advantage of newer devices [3].

Two retrospective studies compared the use of PK energy with CB energy during LRH and LND [16], as well as during LH [31]. Both studies reported significantly shorter operative times in the PK group: 172 min versus 229 min (*p* < 0.0001) in the LRH and LND study [16] and 157.3 min versus 173.4 min (*p* < 0.05) in the LH study [31]. However, a study by Wong et al. stated that LH performed with PK had a statistically significantly longer time to achieve hemostasis than those using LS device (74.4 vs. 63.8 min, *p* = 0.03) [39].

Several studies were found comparing LS to HS [21–23], ENS [40, 41], or conventional bipolar energy [13, 24]. These studies typically reported shorter operative times when using LS.

Two RCTs demonstrated significantly less time required for colpotomy with a monopolar device than HS; however, no differences were observed in total operative times [18, 29].

##### Blood loss

Prompt hemostasis is crucial for the success of surgical procedures. Nearly all studies (36 out of 37) evaluated intraoperative BL as an outcome. ABDv were systematically associated with lower BL in the included studies. Four studies reported significant reductions in intraoperative BL with PK compared to CE [16, 30, 31, 36]. In addition, LS was statistically associated with less BL on LH when compared to UD [21]. However, two RCTs on LH found no significant difference in BL between LS and CB [24] or between LS, bipolar shearer (another ABDv), and HS [25]. Other publications comparing LS with conventional electrical cauterization in LM reported similar results [37].

One RCT on LM conducted by Litta et al. found significantly less BL with HS than CE (δHb: HS = 0.9 vs. CE = 1.2, *p* = 0.004) [27]. In another six studies (including three retrospective studies, two RCTs, and one prospective study), no significant differences between the two devices were found [12, 14, 15, 17, 18]. Conversely, a retrospective comparative study by Huang et al. reported that LS and HS had significantly higher BL than CE on LM (201 vs. 245.8 vs. 175.4 mL, *p* = 0.003) [20].

Regarding BL as an outcome, other studies compared the effectiveness of advanced bipolar devices (including MarSeal™ and ALAN™) and found no statistically significant differences [13, 16, 33, 37–44].

##### Postoperative pain

Limited data are available regarding postoperative pain after laparoscopic gynecological procedures. Only seven studies assessed this outcome, three of which had significant results. Postoperative pain at 24 h was significantly greater with CE in LM and in LRH with bilateral pelvic LND compared to HS and TB, respectively [11, 27]. LS has also been shown to be associated with significant postoperative pain scores in hysterectomy via transvaginal NOTES when compared to CB.

##### Hospital stay length

Several studies assessed the length of hospital stay post-operatively, but only five reported significant results. Three of these studies reported a significantly shorter postoperative stay with HS than CE or LS on LM [20, 27, 28]. Two studies showed significantly shorter postoperative stay with ABDv when compared to CB: LS in a retrospective study on LM (2.10 vs. 2.57 days, *p* < 0.001) [37] (29) and ENS in a RCT on LASH (2.01 vs. 2.17 days, *p* = 0.03) [34].

##### Complications

While most studies focused on perioperative complications, only two retrospective case–control studies reported significant outcomes: one in an oncology setting and another concerning benign gynecologic conditions. The first study found no postoperative complications associated with the PK device used for LRH with bilateral pelvic LND [16]. In contrast, four women in the CB group experienced complications within 60 days post-surgery, including two cases of acute renal failure, two intestinal obstructions, and one vesicovaginal fistula (*p* = 0.02) [16]. However, there was one intraoperative rectal perforation reported in the PK group during the right utero-sacral ligament dissection [16]. In the study conducted by Kuo et al., no complications were noted following a laparoscopic myomectomy using the HS instrument. At the same time, four postoperative complications were recorded in the CE group. These complications included two cases of postoperative fever, one urinary tract infection, and one subcutaneous ecchymosis at the port site (*p* = 0.023) [28].

##### Lateral thermal spread

LTS, a situation characterized by tissue damage some distance away from the place where the active electrode was applied, can occur with all energy sources, leading to some compromise of tissue healing, injury to adjacent structures, and postoperative recovery with potential medicolegal actions associated [3, 6, 45].

Only three studies reported LTS as an outcome, and all compared UD with CE. Choi et al. reported significantly lesser lateral thermal damage during colpotomy with HS than CM instruments (950 μm vs. 1500 μm, *p* = 0.037) [29]. Another study by Gorginzadeh et al. found significantly lesser LTS during colpotomy with HS in the right lateral border (HS = 3.08 mm vs. CM = 3.85 mm, *p* = 0.001) [18].

HS was also reported to allow a more straightforward histologic assessment of the lymph nodes harvested after laparoscopic LND in patients with cervical and endometrial cancer due to a lesser depth of thermal injury in this tissue compared to CE [12].

### Robotic approach

Of the three studies, only one compared several perioperative outcomes between two energy sources. Choussein et al., in a retrospective study on robot-assisted laparoscopic myomectomy (RALM), reported a significantly reduced hospital length of stay with flexible CO_2_ laser fiber compared to ultrasonic scalpel (CO_2_ laser = 0.15 days vs. US = 0.64 days, *p* = 0.004) [46]. No significant difference was found in other perioperative outcomes, such as estimated BL, operative time, or complications.

Two other prospective studies evaluated two different energy sources on the vaginal cuff during robotic assisted total laparoscopic hysterectomy (RATLH) [19, 47]. Billow et al. reported less thermal injury during colpotomy with CO_2_ laser compared to monopolar cautery, where the mean extent of injury using haematoxylin and eosin staining was significantly higher with CM (1.1 mm vs. 0.7 mm, *p* = 0.0191) [47]. Despite Beran et al. showing the feasibility to assess vaginal cuff perfusion using laser angiography during RATLH as the primary outcome, no difference was observed in judged perfusion before or after cuff closure between ultrasonic or monopolar devices [19].

Table 3 summarizes each energy source’s main advantages and disadvantages, as reported across the included studies.

**Table 3:** Main advantages and disadvantages of common energy devices in laparoscopic and robotic gynecologic surgery

Energy source	Advantages	Disadvantages	Notable study findings
Conventional monopolar electrosurgery	Versatility of tissue effects (vaporization, fulguration, desiccation, coaptation) Widespread availability Cost-effective	Potential for stray current injuries Capable of sealing small vessels (< 2 mm) Requires a return electrode (located away from the surgical site) Higher risk for lateral thermal spread	CM was associated with shorter operative time during colpotomy in TLH compared to HS, but resulted in significantly greater lateral thermal damage
Conventional bipolar electrosurgery	Lower voltage required to achieve the desired tissue effect and risk of stray current injury (due to the proximity of the 2 electrodes/jaws); Ability to seal larger vessels vs monopolar devices ($\le$ 5 mm)	Lack of versatility of tissue effects (neither vaporization nor fulguration is possible) Requirement of another device to transect the dissected tissue Limited to vessel sealing ($\le$ 5 mm) Tissue adherence to the electrodes Lateral thermal spread (less vs. monopolar)	CB was as efficient as LS during laparoscopic salpingo-oophorectomy
Advanced bipolar devices (LS, PK, ENS, ALAN, MS, BiCision)	Seals vessels up to 7 mm Feedback-controlled energy Integrated vessel sealing and cutting mechanism Low lateral thermal spread	Costs Availability Bulky jaw in some models	Laparoscopic hysterectomy: ALAN demonstrated the lowest pooled mean operative times ENS was associated with the lowest pooled mean blood loss and shortest hospital stay Laparoscopic supracervical hysterectomy: LS, ENS and BiCision were associated with significantly reduced blood loss, shorter operative time and hospital stay (vs. CB or UD) Laparoscopically assisted vaginal hysterectomy: Pooled mean blood loss, operative time and hospital stay were the lowest using PK Hysterectomy via transvaginal NOTES: LS showed significantly reduced operative time vs. CB (without additional procedures) Laparoscopic myomectomy: the use of LS was associated with a shorter hospital stay compared to PK
Ultrasonic devices	Seal vessels and transect tissues simultaneously Less tissue necrosis and charring Minimal smoke and lateral thermal spread	Slower coagulation (vs. advance bipolar devices) Limited tissue dissection (vs monopolar scissors) Higher post-activation instrument tip temperatures (vs. advance bipolar devices) Blade fatigue	Endometrial and cervical cancer staging: significantly higher number of lymph nodes harvested with HS vs. CE Laparoscopic myomectomy: significantly shorter global operative time, less intraoperative blood loss, postoperative pain score and a shorter hospital stay with HS vs. CE Colpotomy: HS was associated with less lateral thermal damage vs. CM
Thunderbeat™	Combines ultrasonic and bipolar energy Fast cutting and dissecting tissues Seals up to 7 mm vessels High burst pressure Minimal lateral thermal spread	Bulky handpiece Costs	TB was less time-consuming during LRH with lymph node dissection and was associated with less postoperative pain compared to conventional energy
CO_2_ laser	High precision with minimal lateral thermal spread No electrical current through tissue	Costs Availability Training Specialized setup required	Robotic surgery: Total hysterectomy: less lateral thermal spread vs. CM Myomectomy: shorter hospital stay vs. HS

*CB* conventional bipolar, *CE* conventional electrosurgery, *CM* conventional monopolar, *ENS* EnSeal, *LS* LigaSure, *MS* MarSeal, *NOTES* natural orifice transluminal endoscopic surgery, *PK* plasmakinetic system, *TB* thunderbeat

### Risk-of-bias assessment

Most of the identified randomized studies had a high risk of bias, followed by eight studies with a moderate risk. Two RCTs were estimated to have a low risk of bias. Regarding non-randomized studies, almost all were classified as having a high risk of bias, and only three were classified as having a moderate risk.

**Fig. 2:**
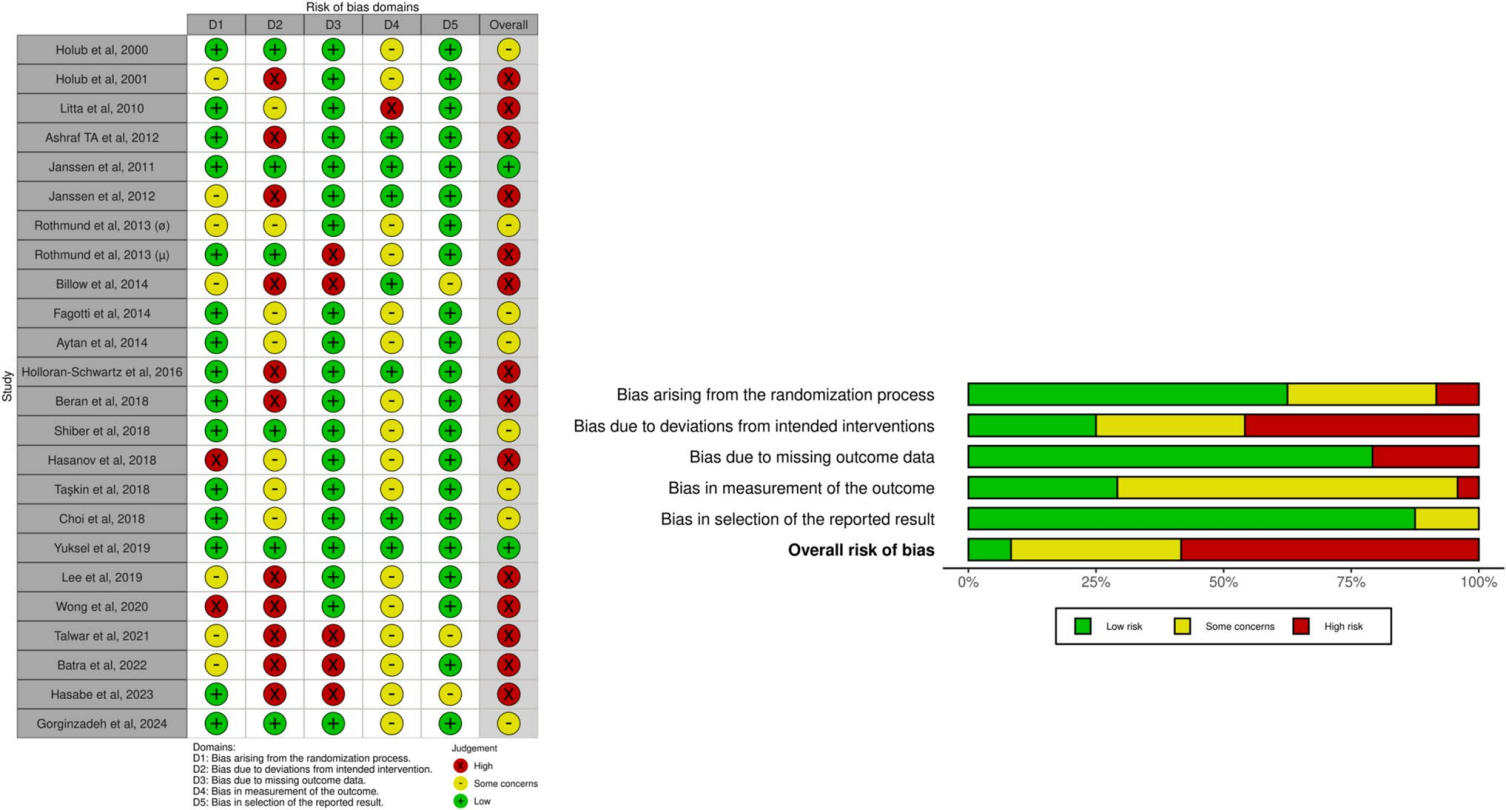
Risk of bias assessment: randomized studies

Figures 2 (randomized studies) and 3 (non-randomized studies) represent the risk-of-bias assessment for all included studies [47]

**Fig. 3:**
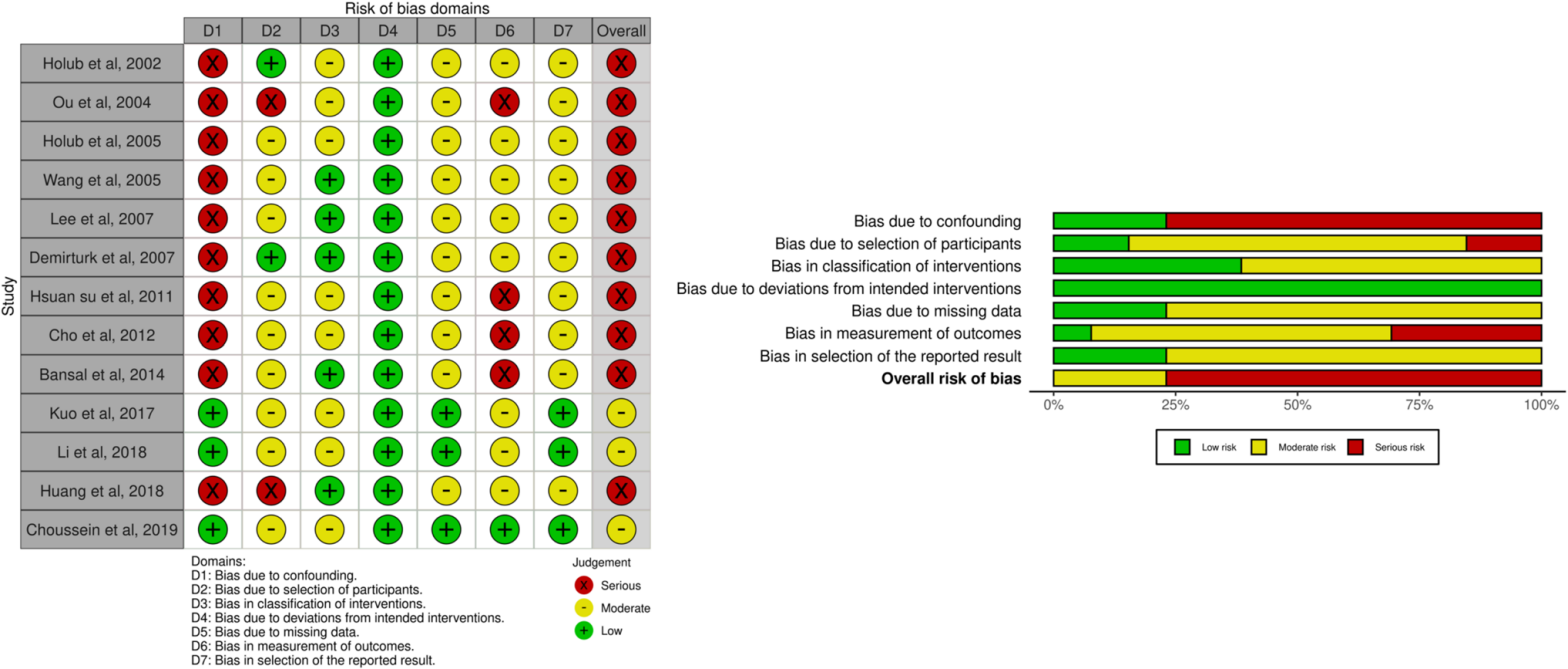
Risk of bias assessment: non-randomized studies

## Discussion

Our review thoroughly examined studies comparing energy sources used in laparoscopic and robotic gynecological surgery. The preference for minimally invasive surgery is continuously increasing over conventional surgery, and the rising popularity of new-generation energy sources makes this review a valuable resource for gynecological surgeons.

The selection and application of energy sources in MIGS are closely linked to the type and complexity of the procedure. While monopolar and bipolar modalities remain the most commonly used, especially in robotic assisted surgery (RAS), their versatility often leads to their integration within multi-modal energy strategies [48]. In clinical practice, monopolar hook electrodes are frequently used for dissection and colpotomy in RAS, while bipolar forceps are applied for coagulation and vessel sealing [48, 49]. The combination of monopolar dissection with advanced bipolar or ultrasonic sealing tools is also common in LH, endometriosis excision, and LND [6, 29, 49, 50]. This multi-energy approach balances precision, hemostasis, and efficiency while minimizing thermal spread [6, 29, 49, 50]. Despite the growing availability of advanced technologies, many surgeons rely on conventional devices due to familiarity, cost-effectiveness, and adaptability to various tissue types [5, 6]

Among all energy devices, UD and ABDv were most consistently associated with shorter operative times in complex laparoscopic procedures, such as those involving LRH and LND in oncological settings. However, the time-saving benefits of these newer devices diminish in standard gynecological procedures, such as salpingo-oophorectomy or hysterectomy, where fewer vascular pedicles need to be sealed [5]. Nonetheless, a surgeon’s familiarity with a specific device is vital in optimizing surgical times [5].

Traditional hemostatic methods utilizing staples and clips have been mainly replaced by electrosurgery [51]. Some studies have reported significantly less BL with ABDv compared to standard energy systems in laparoscopic gynecological surgery [16, 30, 31, 34, 36]. In addition, four studies demonstrated that ABDv had superior hemostatic effects during LH compared to UD [21–23, 26]. Conversely, while some studies showed reduced BL with CE compared to UD, these findings lacked statistical significance. Few studies indicated the advantages of conventional devices over ABDv or HS in terms of hemostasis. However, limitations such as retrospective designs, significant heterogeneity in sample distribution and outcome evaluation, and a lack of routine experience with advanced devices were identified in these studies.

Newer advanced energy devices are associated with less postoperative pain, although data specific to gynecologic laparoscopy remains limited. CB was compared to LS in hysterectomy by transvaginal NOTES, and early postoperative pain was reported to be lower with the first instrument [35]. However, the small sample size in this study and the non-standard nature of transvaginal NOTES in gynecology may complicate the extrapolation of perioperative outcomes.

Most studies reporting significant findings regarding hospital stay length were conducted on LM, where ultrasonic and LS devices demonstrated shorter postoperative stays compared to conventional instruments. Conversely, the ultrasonic scalpel was linked to a significant increase in hospital stay length compared to flexible CO_2_ laser fiber used in RALM [46]. The shorter hospital stays with the latter device may be attributed to its safety profile, resulting from more precise tissue interaction, and the surgical team’s experience with flexible CO_2_ laser fiber and robotic surgery.

The perioperative complication rates among energy sources in gynecologic laparoscopic surgery are generally insignificant. However, two studies reported significant findings, indicating more complications with CE compared to HS during LM or PK during LRH with bilateral pelvic LND [16, 28]. These results may reflect the expected advantages of advanced devices in enhancing surgical safety and efficacy.

There are still limited clinical studies assessing energy-related tissue damage across various energy sources in MIGS. Available laboratory and animal study data suggest that UD is associated with reduced thermal spread in tissues, while monopolar electrosurgery causes the most significant tissue damage [6, 52–55]. Our review included two RCTs reporting similar findings during laparoscopic colpotomy [18, 29]. The reduced operating temperatures required to achieve the desired tissue effect with ultrasonic technology may explain the minimal thermal tissue damage [3, 5, 6]. Conversely, monopolar diathermy can result in higher temperatures and greater lateral thermal spread [3, 5, 6]. In addition, the precision in targeting tissues may explain the reduced thermal injury associated with CO_2_ laser use in colpotomy during RATLH compared to monopolar energy, as noted in a prospective study conducted by Billow et al. [47]. LTS can cause tissue injury, potentially impairing healing and delaying postoperative recovery. The administration of ICG, a safe and widely used fluorescent dye in medicine, can highlight both hypervascularized and hypovascularized tissue, identify poorly perfused areas intraoperatively, and allow for revisions before the end of surgery, ultimately enhancing surgical outcomes [56, 57]. Future studies should incorporate standardized and objective tools for LTS measurement—such as infrared thermal imaging, thermocouple probes, or histological analysis of tissue margins—to support evidence-based selection of surgical energy sources.

The lack of comparative clinical trials on robotic energy sources, particularly in the gynecological context, hinders our ability to address the efficiency differences between devices used in MIGS. Our review is limited by the quality of the included studies, most of which were classified as having a high risk of bias. In addition, recall and selection bias were unavoidable due to the inclusion of non-randomized or retrospective studies. However, the strength of our study lies in the rigorous and comprehensive research methodology applied to the existing evidence by PRISMA guidelines.

In conclusion, introducing advanced energy devices has transformed laparoscopic surgery, enabling more complex procedures due to their optimal thermal and mechanical properties, allowing for the sealing of larger vessels using less energy compared to standard energy systems [6, 51]. It is crucial for surgeons to understand the potential tissue effects associated with each energy source and to select the appropriate device for specific procedures [5, 51]. Therefore, more well-designed studies focusing on various energy sources in MIGS are essential, particularly in the context of RAS, which is on the rise in the gynecological field and where existing literature is limited [58].

## Data availability statement

The data that support the findings of this study are available from the corresponding author upon reasonable request. No datasets were generated or analyzed during the current study.
